# Longitudinal Anti-Müllerian Hormone in Women with Polycystic Ovary Syndrome: An Acupuncture Randomized Clinical Trial

**DOI:** 10.1155/2012/973712

**Published:** 2012-08-26

**Authors:** Jason Franasiak, Steven L. Young, Christopher D. Williams, Lisa M. Pastore

**Affiliations:** ^1^Department of Obstetrics & Gynecology, The University of North Carolina at Chapel Hill, NC 27599-7570, USA; ^2^Reproductive Medicine and Surgery Center of Virginia, Charlottesville, VA 22911, USA; ^3^Department of Obstetrics & Gynecology, University of Virginia, P.O. Box 800712, Charlottesville, VA 22908-0712, USA

## Abstract

Others have studied acupuncture treatment for polycystic ovary syndrome (PCOS). Anti-müllerian hormone (AMH) is positively correlated with the ovarian follicle pool, thus making it a useful ovarian reserve measure. AMH is elevated in women with PCOS and has been suggested as a diagnostic tool. This study examined the impact of electroacupuncture on AMH concentration in women with PCOS. Seventy-one women with PCOS participated in a randomized, double-blind, sham-controlled clinical trial of acupuncture. Three longitudinal AMH samples over the 5-month protocol were compared with objective ovulation parameters primarily using nonparametric statistics. Results indicated that AMH levels in PCOS were higher than published norms in women without PCOS. There was no difference between the true and sham acupuncture arms in the change in AMH longitudinally. Baseline AMH, but not the change in AMH over time, was inversely correlated with ovulation and menstrual cycle frequencies in both arms combined (*P* < 0.001). In conclusion, AMH correlated with an increased likelihood of monthly ovulation, as expected from the literature on women without PCOS. The lack of difference by intervention in AMH was consistent with the underlying clinical trial. AMH may be clinically useful to predict which PCOS women are more likely to respond to an intervention.

## 1. Introduction

Polycystic ovary syndrome (PCOS) is the most common endocrine disorder in women of reproductive age with incidence ranging from 8.7% to 17.8% depending upon which criteria are used [1]. Diagnosis of PCOS is made when androgen excess, ovulatory dysfunction, and/or polycystic ovaries are identified after the exclusion of other disorders that can cause these signs and symptoms [2]. Although not a part of the formal diagnosis, a number of endocrine abnormalities are often seen, including an increased ratio of luteinizing hormone (LH) to follicle stimulating hormone (FSH) [3] and insulin resistance [4]. There is emerging evidence that serum anti-müllerian hormone (AMH) may be useful in the diagnosis of PCOS [5].

AMH is expressed by the ovarian preantral and early antral follicles and reflects the size of the follicular pool. AMH levels gradually decline as the follicle pool declines, and it is thus useful as a marker of ovarian reserve, although there is no consensus on a threshold value for diagnosis of diminished ovarian reserve [6–8].

Emerging evidence has linked elevated AMH levels with women who have PCOS. AMH concentration correlates well with clinical, endocrine, and ultrasound markers associated with PCOS and may be a useful marker for the extent of disease [5, 9]. Although growing data support AMH as a possible clinical diagnostic and/or prognostic marker, there is little known about changes in AMH level in response to an intervention.

Acupuncture has been shown to be efficacious for a number of medical conditions. Of women seen in a reproductive endocrinology and infertility clinic, 22% had tried acupuncture therapy within 18 months of their initial clinic visit in the USA [10] and 12.5% within 6 months in Australia [11]. Researchers found an 8% use of acupuncture among infertility patients in the UK [12]. Interestingly, we found no difference in ovulation rates or ovarian hormones levels between the active acupuncture and sham acupuncture arms of a randomized clinical trial, although there was a suggestion that both arms benefited from improved frequency of ovulation during the study [13].

Given these results, we set out through a secondary analysis of a randomized clinical trial to determine if AMH levels can predict response to acupuncture and/or can predict ovulation among oligoovulatory and anovulatory untreated, adult female patients with PCOS. Additionally, we aimed to characterize the levels of AMH of this subset of women relative to women without PCOS by age, as reported by others.

## 2. Materials and Methods 

### 2.1. Population

This is a secondary analysis of AMH results from a randomized, double-blind, sham-controlled clinical trial of acupuncture in women diagnosed with PCOS. The original trial is described in detail elsewhere [13]. In summary, the 5-month protocol involved baseline questionnaires and biological sampling, 2 intervention months, post-intervention repeat questionnaires and biological sampling, 3 months of follow-up without intervention, and post-follow-up questionnaires and biological sampling. Women provided urine or blood samples weekly throughout the entire 5 months for objective assessment of ovulation. Menses were self-reported. This trial was approved by the University of Virginia's Institutional Review Board (no. 12045).

Inclusion criteria were (a) a diagnosis of PCOS, as confirmed by the presence of both oligomenorrhea and hyperandrogenism per the US National Institutes of Health criteria [14], (b) aged 18 to 43 years, (c) at least one menses in the past six months but no more than eight periods in the most recent 12 months without hormonal intervention, and (e) agreement to not take hormonal contraceptives, metformin, or fertility medication for the 5 months of study participation. Exclusion criteria were (a) diagnosed with Cushing's Syndrome, uncontrolled thyroid disease, hyperprolactinemia, congenital adrenal hyperplasia, and diabetes mellitus, (b) use of metformin or hormonal contraceptives in the 60 days prior to enrollment, (c) use of any other hormonal drug in the 30 days prior to entry into study, including fertility medications, and over-the-counter hormonal supplements or herbs (i.e., black cohosh, clover, soy, dong quai/Chinese angelica root, fructus rubi, and white peony root), (d) currently pregnant or breastfeeding during the prior 30 days, (e) any acupuncture treatment for ovulatory disorders in the prior 30 days, (f) weight > 113.4 kg (250 pounds), (g) currently taking anticoagulation medication other than low-dose (≤81 mg) aspirin, (h) immune deficient, and (i) history of any bleeding disorder.

### 2.2. Interventions and Ovulation Assessment

Subjects were randomized to 12 acupuncture or sham sessions: twice each week for the first four weeks followed by once per week for an additional four weeks. For the true acupuncture treatment, the following bilateral points were stimulated with electroacupuncture (EA): Bladder 23, Bladder 28, Spleen 6, and Spleen 9. The following points were manually stimulated: Pericardium 6, Triple Energizer 5, and Governor Vessel 20. The sham acupuncture was performed with the validated Park Sham Device [15, 16]. The sham device was placed on the skin at standardized points on all four extremities (Achilles tendon and lateral head of the triceps) chosen in order to avoid standard acupuncture meridians and acupuncture points [17]. For further details, the reader is referred to a prior publication [13]. 

The participants provided weekly blood samples for serum progesterone measurement or collected first-void urine samples at home (stored in their home freezer) for pregnanediol glucuronide (PDG) measurement, for the entire 5-month protocol. Ovulation was defined as progesterone ≥3 ng/mL or a ratio of the peak urinary PDG to the basal PDG level in the follicular phase ≥4.0. 

### 2.3. AMH Assays

Serum AMH was measured longitudinally on all study participants using samples collected at their three study center visits (preintervention, postintervention, and after 3 months of follow-up). The assays were conducted by the Clinical Laboratory Research Core at Massachusetts General Hospital (Boston, MA, USA) using an AMH Gen II ELISA kit from Beckman Coulter according to the manufacturer's protocol. The sensitivity of the assay was 0.05 ng/mL.

### 2.4. Statistical Analyses

Medians and interquartile ranges were calculated by intervention arm, rather than means and standard deviations, due to the modest sample size. Graphs were created to investigate the distributions. Potential differences between the intervention arms were assessed with Kruskal Wallis, Wilcoxon Rank Sum, or Sign tests (continuous variables) and Spearman chi-square tests (categorical variables). After zero-skewness log transformation of AMH, linear regression was used to develop lines-of-best fit. *t*-tests were used to compare this cohort to the literature. Power calculations were not run *a priori* due to the fact that this was a secondary analysis. Statistical significance was judged by a two-sided alpha ≤ 0.05, unless otherwise specified. All statistical analyses were conducted with STATA/IC 12 software (STATA Corp, TX, USA).

## 3. Results

**Figure 1:**
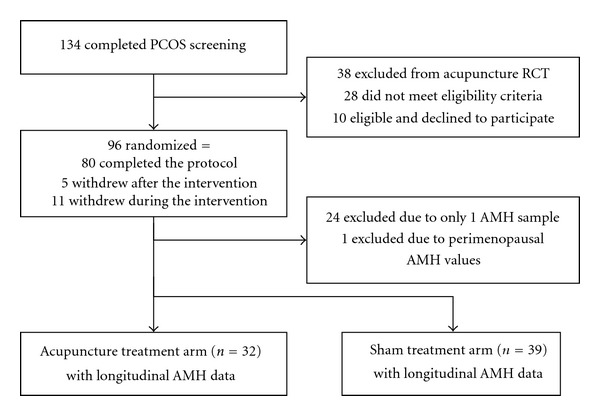
Flowchart of study participation.

Ninety-six women were eligible, consented, and were randomized for the underlying acupuncture clinical trial [13], and 72 had more than one AMH sample as required for this longitudinal analysis. Of those 72 women, one was subsequently dropped from the dataset due to perimenopausal AMH levels (<0.05 mg/mL) despite a normal FSH level at baseline (2.7 mIU/mL) (Figure 1). Of the final cohort of 71 women diagnosed with PCOS, 32 received active acupuncture and 39 received sham acupuncture. These women were randomized between February 2006 and August 2009.

Most of the women had some college education and were Caucasian (Table 1). The median body mass index was 29-30 kg/m^2^. There were no differences by intervention in age, education, BMI, race, or Hispanic ethnicity (*P* > 0.10). Selected eligibility data and endocrine results are also displayed in Table 1. On average, the participants had 5-6 menses in the most recent 12 months without hormonal intervention before enrollment. 

The preintervention AMH concentration did not vary between the intervention arms (*P* = 0.79, Table 2), nor were there differences in AMH by intervention at the other two time points (*P* > 0.80). The change in the AMH concentration was not clinically relevant by intervention arm. AMH increased by 0.02 and decreased by 0.005 ng/mL in the true and sham arms, respectively, after the intervention (*P* > 0.30). AMH decreased by 0.05 and increased by 0.01 ng/mL in the true and sham arms, respectively, after the entire 5-month protocol (data not displayed, *P* > 0.40).

No differences were detected between the true and sham acupuncture interventions in terms of the relationship between AMH concentration and both ovulation and menses. There was no correlation between the pre- and postintervention change in AMH level and the ovulatory frequency or menstrual cycle frequency in either the acupuncture arm (*P* > 0.30) or the sham arm (*P* > 0.50). Similarly, there were no corresponding correlations when the timeframe was expanded to include the entire 5 months of the protocol (*P* > 0.30). The preintervention AMH concentration did not predict who would become a “responder” to the true acupuncture, defined as at least a 60% monthly ovulation rate over the entire study timeframe (*P* = 0.29). The change in the AMH concentration over the 5-month protocol was not associated with being a “responder” in the true acupuncture arm (*P* = 0.66).

Combining the intervention arms in Table 3, the preintervention AMH concentration was significantly inversely related to both the ovulatory frequency during the trial (Spearman's *r* = −0.54, *P* < 0.001) and the frequency of menses during the trial (Spearman's *r* = −0.50, *P* < 0.001). Combining the intervention arms, there was no correlation between the change in AMH level either during the intervention only or the entire 5-month trial and the ovulatory frequency or menstrual cycle frequency (*P* > 0.55).

**Figure 2:**
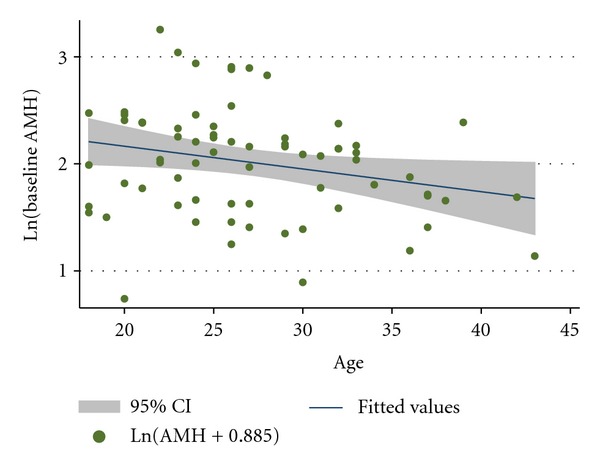
Preintervention log-transformed AMH concentration by age in PCOS women.

Age approached statistical significance in terms of being associated with the preintervention AMH levels in this PCOS cohort (*P* = 0.053).  Figure 2 displays the log-transformed AMH concentration by age with a line-of-best-fit, which corresponds to the equation

(1) $$\begin{array}{c} \text{Ln}\;\;\left({\text{AMH}}_{0}\right)=2.6-0.02\;\;\left(\text{Age}\right). \end{array}$$

**Figure 3:**
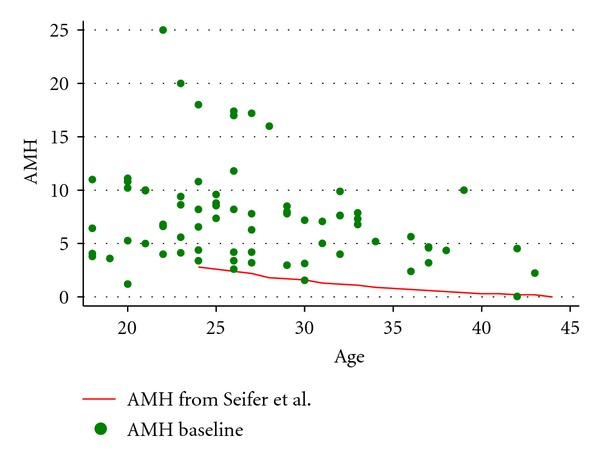
Preintervention AMH concentration (ng/mL) by age in PCOS women and compared to Seifer et al.'s [6] population of 17,120 women.

The AMH concentration by age was significantly higher in the PCOS cohort (*P* < 0.0001) in comparison to a cohort of 17,120 women from infertility clinics across the USA [6]. The comparison cohort is graphed as a heavy red line in Figure 3. For comparison purposes, the equation underlying the line-of-best-fit for this cohort from Seifer et al's is

(2) $$\begin{array}{c} {\text{AMH}}_{\text{Seifer}}=6.21-0.15\;\;\left({\text{Age}}_{\text{Seifer}}\right). \end{array}$$

AMH was not related to BMI (Spearman's *P* = 0.45). The median AMH by BMI tier is as follows: 6.4 for 20.0–24.9 BMI, 8.4 for 25.0–29.9 BMI, 7.6 for 30.0–34.5 BMI, 7.5 for 35.0–39.9 BMI, and 4.3 for 40.0–44.9 BMI.

## 4. Discussion

### 4.1. Summary

Our investigation revealed that this acupuncture protocol and this sham protocol individually did not significantly alter the serum AMH concentrations in this cohort of women with PCOS. Combining the two protocols, the pre-intervention AMH concentration was positively correlated with both the ovulation frequency and the menstrual cycle frequency during the 5-month clinical trial protocol (*P* < 0.0001). The AMH concentration was significantly higher across all ages in the PCOS cohort in comparison to a very large cohort of fertility clinic patients (*P* < 0.001).

### 4.2. Comparison to Relevant Literature

Low serum AMH levels have been shown to be predictive of infertility treatment in women without PCOS. Lower serum AMH concentrations during assisted reproduction treatment are strongly associated with a reduced oocyte yield and low oocyte quality [18, 19]. For women with PCOS who had AMH levels that were 2 to 3 times the level produced by normal ovaries [20] the opposite appeared to be true in that women with high serum AMH predicted poor response to treatment [21, 22]. It was not surprising that the AMH concentrations were not associated with the real or sham acupuncture protocols, as the underlying randomized clinical trial found that there was no difference in the ovulation rate by intervention arm [13]. We predicted a decline in the AMH concentrations toward non-PCOS levels in women who responded to the intervention; however, any decline was not statistically significant. In one study with 65 PCOS cases, serum AMH concentrations declined an average of 7% after six months of metformin treatment (*P* < 0.01) [23]. A 7% decline in our measurements would have corresponded to a half point. 

Preintervention serum AMH concentrations predicted success in terms of regular ovulation and menses, as has also been reported in women without PCOS [18, 19]. This finding suggests that AMH levels could serve as a marker of the likelihood of PCOS resolution without treatment or may support the underlying clinical trial's original report suggesting that both interventions provided benefit [13]. One prior report did find that baseline AMH levels in women with PCOS predicted the response to treatment; in their case the treatment arms were laparoscopic ovarian drilling and clomiphene citrate (*P* < 0.01, [24]).

As expected, there was an inverse association between age and AMH concentration, as reported by others [5, 23, 25, 26]. If we were to impose a linear structure on our AMH values in order to compare to the literature, then the slope of the AMH/age association in our PCOS cohort was −0.19. This slope was somewhat steeper than a prior report (−0.11 [25]) and was surprisingly similar to the slope of the AMH decline among women without PCOS who were seen in a fertility clinic (−0.15) [6]. This implies that the rate of decline in AMH as a woman ages is similar in both PCOS and non-PCOS populations. One area of future research, unrelated to the acupuncture intervention, is a description of the AMH concentrations in women with PCOS as they progress through the menopausal transition to their final menstrual period.

Most of the prior research did not find an association between AMH and BMI in women with PCOS [9, 23, 25], although one publication did report an inverse correlation [26]. The varying results in the literature on this item may be due to different presentations of PCOS as reported by Lin et al. in 2011 [26] or may be due to serum versus plasma AMH measurements.

### 4.3. Strengths and Limitations

The findings are limited by the fact that the acupuncture protocol did not allow for individualization, thus the findings are not reflective of “real world” acupuncture practice. Therefore, the interpretation of the results is limited to this particular acupuncture (and sham) protocol. There was no consideration of phenotypes within the PCOS diagnostic framework, as was investigated by others [26]. The sham procedure might not have been inert, as the underlying clinical trial results implied a benefit to both the true and sham acupuncture groups. Thus there may be a true impact of acupuncture on AMH, which might have been observed if the comparison had been an observation-only cohort. Our sample was also limited to women with longitudinal AMH samples, thus women who became pregnant or dropped out of the study during the 2 months of the intervention (*n* = 13) could not be included, and this may have influenced our findings if those women had lower AMH levels than the median of the population analyzed.

A strength of this study is the RCT design, with its prospective data collection and blinding of the intervention. This is the only clinical trial of acupuncture to measure AMH in women and one of only a few reports [23, 24] with longitudinal assessments of AMH in a cohort of women with PCOS. The diagnosis of PCOS was conducted objectively through this study on all women, thus avoiding bias at the time of the eligibility assessment. Ovulation was measured objectively through serum progesterone and urinary pregnanediol glucuronide, as reported previously [13]. This study included PCOS women from the general community (approximately 40% had not been diagnosed with PCOS prior to study enrollment) as opposed to a clinic population with potentially more severe disease. The cohort represented a wide range of ages and BMI, which improves the generalizability of our findings.

### 4.4. External Validity

These findings are applicable to women with PCOS as diagnosed with NICHD criteria. As stated above, the interpretation of the results is limited to this particular acupuncture (and sham) protocol.

### 4.5. Clinical Significance

In women without PCOS, AMH has been increasingly used as a marker of ovarian reserve [27] and has been shown to be a potential predictor of success in assisted reproduction [18, 19]. AMH has been shown to be elevated in women with PCOS [20] and may become a useful marker of PCOS. Our study showed elevated levels of AMH, consistent with other reports [5, 18, 20, 21], and it appears that this elevation may be an arithmetic deviation (as opposed to a multiplicative relationship) that is fairly consistent over a wide age range. Importantly, preintervention AMH levels were associated with ovulatory rates during the course of the trial, lending further evidence that AMH could become a potential marker for severity of disease in women with PCOS.

AMH concentrations were not associated with the real or sham acupuncture protocols consistent with the findings from the underlying randomized clinical trial which found no difference in the ovulation rate by intervention arm [13]. Thus, we were unable to define clinical or serum markers associated with successful treatment with this acupuncture protocol in women with PCOS. More research is indicated in this particular area of fertility treatment of women with PCOS.

## Figures and Tables

**Table 1:** Participant demographics and biochemical data by intervention arm.

Factor	True acupuncture (*n* = 32)	Sham acupuncture (*n* = 39)	*P* value
Age: median (IQR)	27.5 (22–33)	25 (23–29)	0.14
Education: *n* (%)			
HS or less	2 (6%)	3 (8%)	0.89
Some college	14 (44%)	15 (38%)	
College degree	7 (22%)	13 (33%)	
More than college	9 (28%)	8 (21%)	
Body mass index: Median (IQR)	29.3 (23.5–36.3)	29.9 (24.4–34.9)	0.99
Race: *n* (%)			
Caucasian	25 (78%)	32 (82%)	0.67
African-American	3 (9%)	4 (10%)	
Other	4 (13%)	3 (8%)	
Hispanic: *n* (%)	0 (0%)	3 (8%)	0.11
Menses in the 12 months prior to enrollment without hormonal medications	6 (3.5–7)	5 (3–7)	0.31
Fasting plasma glucose (mg/dL): median (IQR)	93 (88–96)	94 (89–98)	0.62
Fasting serum insulin (mIU/mL): median (IQR)	7.8 (3.5–13.3)	6.9 (2.7–10.9)	0.46
TSH (uIU/mL): median (IQR)	1.36 (0.84–1.91)	1.51 (1.07–2.02)	0.50
17 OHP (ng/dL): median (IQR)	121 (81–148)	124 (76–150)	0.62
HbA1C: median (IQR)	5.3 (5.1–5.5)	5.3 (5.1–5.6)	0.34
DHEAS (*μ*g/dL): median (IQR)	129 (101–231)	174 (126–214)	0.75
Free testosterone (pg/mL): median (IQR)	11.3 (7.6–14.6)	11.1 (7.6–18.7)	0.67
SHBG (nmol/L): median (IQR)	33.1 (21.7–58.0)	33.5 (23.0–53.4)	0.90

**Table 2:** Longitudinal AMH concentrations (ng/mL) in women with PCOS by intervention: median (interquartile range).

Intervention arm	Preintervention baseline	Postintervention	*P* value^a^	Three-month follow-up	*P* value^a^
True acupuncture (*n* = 32)	6.5 (4.4–9.9)	6.4 (4.5–10.9)	0.36	6.2 (5.0–9.2)	0.57
Sham acupuncture (*n* = 39)	7.4 (4.1–9.6)	6.4 (4.5–9.0)	0.63	5.8 (4.2–10.4)	0.43
*P* value^b^	0.79	0.90		0.84	

^a^One-sided test for a decline in AMH since the preintervention value.

^b^Two-sided test comparing true versus sham acupuncture.

**Table 3:** Correlation between AMH concentrations and both ovulation and cycle frequency for the entire PCOS cohort: Spearman's rho (*P* value).

AMH variable	Ovulatory frequency	Menstrual cycle frequency
AMH preintervention	−0.54^∗^ (<0.0001)	−0.50^∗^ (<0.0001)
Change in AMH post- versus preintervention	−0.02 (0.90)	−0.07 (0.58)
Change in AMH 3 month follow-up versus preintervention	0.08 (0.53)	−0.01 (0.92)

^∗^ *P* ≤ 0.05.
